# Editorial: Investigating exposures and respiratory health in coffee workers

**DOI:** 10.3389/fpubh.2022.1026430

**Published:** 2022-09-08

**Authors:** Mohammed Abbas Virji, Kristin J. Cummings, Jean M. Cox-Ganser

**Affiliations:** National Institute for Occupational Safety and Health, Centers for Disease Control and Prevention, Morgantown, WV, United States

**Keywords:** coffee workers, respiratory diseases, α-diketones, dust, engineering controls intervention

Workers in the coffee industry face a variety of inhalational hazards. These range from predominantly organic dust, endotoxin, and green and castor bean allergen exposures in the primary processing factories to dusts, gases, and vapors including α-diketones in coffee production facilities (1–5). Previously documented respiratory health effects include symptoms such as wheeze, cough, and dyspnea, bronchial hyperresponsiveness, reduced spirometric parameters, and chronic lung diseases including asthma and obliterative bronchiolitis (OB) (3, 5–10). Some of these studies are decades old, while some are notable for small size and limited exposure assessments. In this special issue of *Frontiers in Public Health* on “Investigating exposures and respiratory health in coffee workers”, a series of articles explores in detail the exposures, emissions, engineering controls, and health consequences across the contemporary coffee industry by describing studies of primary processing in 16 factories in two African countries and coffee production in 17 facilities in the United States.

The article by Bratveit et al. summarizes exposures, health effects and exposure-response relationships in a combined dataset of cross-sectional studies conducted in the previous decade in primary coffee processing factories in Tanzania and Ethiopia. At these factories, green coffee beans are cleaned, hulled, sorted and packaged for shipping. High levels of organic dust and endotoxin exposures were measured that frequently exceeded their respective occupational exposure limits. They also report increased prevalence of chronic respiratory symptoms, lowered forced expiratory volume in 1 s (FEV_1_) and forced vital capacity (FVC) that were significantly associated with cumulative organic dust exposures in male workers. They also highlight the importance of increasing health and safety knowledge and competency among health personnel, politicians, and stakeholders for prevention of occupational injuries and diseases in these two developing countries.

Other articles in this issue are based on data collected by the U.S. National Institute for Occupational Safety and Health (NIOSH) at coffee production facilities (11). In these facilities, managers and employees were primarily concerned about the risk of OB in relation to exposure to α-diketones, especially in light of the recommended exposure limits (RELs) for diacetyl and 2,3-pentanedione established by NIOSH in 2016 (12). Previous studies had demonstrated adverse respiratory effects among workers exposed to α-diketones in workplaces manufacturing or handling flavoring chemicals or flavored food products (13).

In the NIOSH evaluations, extensive exposure assessments were conducted for diacetyl, 2,3-pentanedione and volatile organic compounds during coffee handling, roasting, grinding, flavoring, packaging, shipping and work in quality control and cafés (LeBouf, Blackley et al.). These data were used to evaluate exposure determinants and emissions factors to facilitate prioritization of exposure mitigation and to generate metrics of peak, average, and cumulative exposure for epidemiologic analysis (Virji, Cummings et al.; LeBouf, Ranpara et al.). An innovative approach was taken to model diacetyl and 2,3-pentanedione exposure determinants using Bayesian mixed models and a Bayesian model averaging method (Blackley et al.). The authors identified determinants with higher exposures such as grinding or open storage of coffee beans, which may be amenable to modification, and those with low exposures such local or general exhaust ventilation, whose use can be encouraged. They highlight some challenges including effectively assessing complex mixtures of chemicals, historical exposure characterization, and collecting more refined exposure determinants.

The health assessment articles report a range of upper and lower respiratory symptoms, respiratory abnormalities and asthma in these coffee production workplaces, including a case of OB in a worker exposed to flavoring chemicals (Harvey, Fechter-Leggett et al.; Harvey, Blackley et al.). The authors suggest that the patterns of symptoms and lung function abnormalities may be indicative of early disease markers or subclinical disease. Decrements in percent predicted FEV_1_ and FVC and small airway abnormality on impulse oscillometry were associated with various metrics of exposure to diacetyl, 2,3-pentanedione and the sum of the two α-diketones (Virji, Fechter-Leggett et al.). These effects were strongest among flavoring workers but were also observed in non-flavoring workers. Although the health assessment and exposure-response analysis were extensive, the authors report certain challenges and limitations such as modeling mixed exposures, potential for healthy worker survivor effect, recruitment bias, and few cases of abnormal spirometry.

The article by Johns et al. discusses the impact of various factors and assumptions of risk assessment such as the choice of health effect, use of human or animal studies, quality of exposure assessment, inter-species extrapolation and uncertainty factor that have resulted in a wide range of suggested exposure limits. The authors emphasize the need for transparency in assumptions and methods used to understand the variability in the proposed exposure limits. While additional data are gathered to fill in knowledge gaps in risk assessment, mitigating exposures to α-diketones in coffee production offers the best opportunity to prevent adverse respiratory health outcomes (Stanton et al.) (14). Indeed, the findings of Stanton et al. demonstrates that installing ventilated enclosure on grinding equipment significantly reduced α-diketone exposures near grinders by 75–95%, and in the rest of the facility by 15–61%. Installing engineering controls was also recommended in the study of primary coffee processing.

In both the coffee processing and production studies, standardized data collection enabled data aggregation, facilitating the detection of exposure-response relationships that were otherwise inconsistent between Tanzania and Ethiopia or may not have been observed in individual U.S. production facilities. Given the large number of small- to medium-sized facilities across most industries, these studies highlight the benefits of standardizing data collection and data pooling to increase sample size and the power to detect subtle exposure-response relationships, achieve a more representative population, and make robust inferences (Virji, Cummings et al.). Indeed, aggregating data across multiple industrial, occupational or disease cohorts has long been conducted to take advantage of increased population size (15–17). There are numerous examples of such epidemiologic data aggregation, a vast majority of which are done in a *post-hoc* manner (18), but include some *a priori* aggregation planned in the study design phase (19). *A priori* aggregation is most desirable because a common approach is used to collect data which minimizes differences among studies.

There are likely overlapping health effects of different respiratory hazards that coffee production workers are exposed to. Similar clinical and functional effects can occur in asthma, OB and other chronic respiratory diseases, making it difficult to distinguish among health outcomes. Advanced machine learning methods now make it feasible to explore underlying patterns in multiple symptoms and lung function tests that may help to classify workers into groups indicative of different health outcomes or different stages of disease (20). Such methods may identify early disease stage which can enable timely intervention to prevent the development or progression of lung disease and protect co-workers.

Modeling exposure-response relationships for mixed exposures is challenging, despite efforts to bring attention, resources, and tools to address mixtures (21). Single chemical models of chemical mixtures do not represent the workplace reality, and statistical approaches may not adequately address highly correlated exposures in the same model. Alternatively, multiple chemicals can be combined to generate an aggregate value based on simple addition as was done in the NIOSH study or taking into consideration quantitative structure-activity relationships.

The success of these studies is in part attributed to the well-planned, standardized data collection combined with comprehensive, high-quality health and exposure assessments that led to robust results. The articles in this series enhance knowledge of exposure-response relationships for α-diketones and show efficacy of well-designed controls. A highlight of this research is the integration of exposure and health characterization for evaluating exposure determinants and risk factors for adverse health outcomes, risk assessment tools, and the efficacy of engineering controls. This approach fits within the paradigm of translational research framework for environmental health science aimed to maximize public health benefit of research studies (22, 23). Such an integrated approach can lead to more accurate health risk estimates and appropriate and targeted exposure mitigation recommendations, ultimately resulting in a reduction in the burden of adverse respiratory health outcomes for workers.

## Author contributions

MV, KC, and JC-G contributed to the conception and design of the study. MV wrote the first draft of the manuscript. All authors contributed to manuscript revision, read, and approved the submitted version.

## Conflict of interest

The authors declare that the research was conducted in the absence of any commercial or financial relationships that could be construed as a potential conflict of interest.

## Publisher's note

All claims expressed in this article are solely those of the authors and do not necessarily represent those of their affiliated organizations, or those of the publisher, the editors and the reviewers. Any product that may be evaluated in this article, or claim that may be made by its manufacturer, is not guaranteed or endorsed by the publisher.
